# Malignant neoplasia arising from ovarian remnants following bilateral salpingo-oophorectomy (Review)

**DOI:** 10.3892/ol.2014.2089

**Published:** 2014-04-25

**Authors:** ATSUSHI IMAI, KAZUTOSHI MATSUNAMI, HIROSHI TAKAGI, SATOSHI ICHIGO

**Affiliations:** Department of Obstetrics and Gynecology, Matsunami General Hospital, Gifu 501-6062, Japan

**Keywords:** ovarian remnant syndrome, serous cystadenoma, clear cell carcinoma, endometrioid adenocarcinoma, endometriosis

## Abstract

Ovarian remnant syndrome (ORS) is a rare, but well-known gynecological complication, most often induced by difficult bilateral salpingo-oophorectomy (BSO) procedures that leave residual ovarian tissue on the pelvic wall. The most common preexisting conditions for this complication include endometriosis, pelvic inflammatory disease and prior abdominal surgery. The residual ovarian tissue may eventually cause malignant development. A total of 12 cases of malignant and benign tumors (clear cell adenocarcinoma in 1 case, mucinous-type tumors in 2, endometrioid-type tumors in 5, adenocarcinoma in 3 and border serous neoplasia in 1) and 21 benign cysts developing from an ovarian remnant have been described in the literature to date. Endometriosis, known to increase the risk of ovarian cancer, predisposes patients to ORS, with an incidence rate of 30 to 50% in ORS patients with ovarian carcinoma. Although the true incidence of ORS remains unknown, when endometriotic adhesions are diagnosed during BSO, the possibility of ORS and subsequent ovarian malignant transformation may mandate complete surgical resection.

## 1. Introduction

Ovarian remnant syndrome (ORS) is defined as locating histologically-confirmed ovarian cortical tissue during explorative surgery in a female patient who had previously undergone bilateral salpingo-oophorectomy (BSO) and now presented with a pelvic mass or pain (1–10). ORS usually occurs as a result of incomplete tissue removal. Endometriosis, pelvic inflammatory disease or previous gynecological surgeries have been known to increase the risk of ORS, as the removal of ovarian tissue is made more difficult by dense fibrotic adhesions that become more likely between an ovary and the surrounding structures. With an increase in the number of laporoscopic ovarian surgeries performed, implantation of ovarian tissue to ectopic sites (e.g., the trochar site, port site and abdominal wall) has also been recognized as a significant cause of ORS (8,9,11–13). Case studies on the syndrome have become more common over the past several years, although the true incidence of ORS remains unknown. This increase in reported cases is likely to be as doctors have become more aware of the condition and imaging techniques are more readily available (13).

Malignant and benign tumors may be found within ovarian remnant tissues. In the literature to date, 12 cases of primary ovarian cancer and 21 cases of benign cysts developing in the ovarian remnant have been described. The present review aims to evaluate the clinical features and pathological findings of ovarian tumors developing in ovarian remnants following BSO.

## 2. Histological types

Histological examination of ovarian remnants has revealed a wide range of results, including follicular cysts with or without hemorrhage, endometriosis and the presence of a corpus luteum (1–4,6,13–15). Neoplasia is a rare finding in ORS. In the literature, 12 cases of adenocarcinoma and border malignancy developing in an ovarian remnant have been described to date (Table I) (6,14,16); 1 case of clear cell adenocarcinoma, 2 of mucinous tumor types, 5 of endometrioid types, 3 of adenocarcinomas and 1 of border serous neoplasia.

The high prevalence of ovarian endometriosis among cases of malignant development in ORS raises a permanent issue that has been increasingly debated in recent times; the link between endometriosis and ovarian cancer (14,17). The incidence of endometriosis in females with ovarian cancer is between 8 and 30% (5,18,19). Kho and Abrao highlighted that endometriosis predisposes patients to ORS and is associated with 50% of patients with ovarian carcinoma (4). Malignancies of endometrioid and clear cell histology provide the greatest risk of developing ORS (20–22). In a study by Brinton *et al* (22), in Denmark between 1978 and 1988, a population-based cohort of females was evaluated. Although the association was restricted to endometrioid [relative risk, 2.53; 95% confidence interval (CI), 1.19–5.38] and clear cell (relative risk, 3.37; 95% CI, 1.24–9.14) malignancies, it was determined that females with endometriosis had a predisposition to developing ovarian cancer. Similarly, in a study that interviewed 812 females diagnosed with ovarian cancer, Rossing *et al* (19) found that the risk of endometrioid/clear cell ovarian cancer for patients with endometriosis was three-fold greater compared with population-based controls. By contrast, there was no increase in the risk associated with other ovarian cancer histological sub-types.

Benign serous neoplasia arising from persistent ovarian remnants has been documented in 5 cases (Table I) (10,15). These studies highlight the neoplastic potential of ovarian remnants and further discuss the available data on the risk of malignant transformation, as spontaneous resolution of a neoplasm is unlikely and there is evidence for malignant transformation of certain benign serous tumors. Between 10 and 15% of serous cystadenomas show atypical serous epithelial proliferation with hyperchromic and rounded nuclei and evident nucleoli. The transition from benign to malignant epithelium in serous ovarian tumors has been described by Puls *et al* (23). The review of 96 cases of ovarian serous and mucinous cystadenocarcinoma, lead to the observation of benign epithelium adjacent to an area of borderline or malignant epithelium in 74 tumors (79%), and a site of epithelium transition was noted in 38 (40%). These findings are in agreement with epidemiological and molecular genetic data indicating that there is potential for malignant transformation in certain benign serous or mucinous ovarian tumors. As indicated by Mahdavi *et al* (15), ovarian cancer may be prevented in these cases by removal of the tumors, particularly in postmenopausal patients.

### Time to ORS

To date, there has been no clear data regarding the time interval between ORS and neoplasia found within ovarian remnants. The mean time to the development of adenocarcinoma in ovarian remnants is 12.6 years (range, 2–54 years) after previous surgery.

## 3. Incidence

The incidence of adenocarcinoma and cystadenoma in ovarian remnants cannot be realistically calculated from the current small number of published series. Among the 186 patients included in the largest study by Magtibay *et al* (2), no cases of adenocarcinoma were encountered following excision of the ovarian remnants. The first case of ORS following laparoscopic salpingo-oophorectomy was described by Nezhat *et al* (13), however, no adenocarcimona was observed during follow-up. Furthermore, Kho *et al* (6) reviewed the outcomes and pathological findings of 20 cases of ORS, and 2 were found to exhibit malignancies in the remnant ovarian tissue. The incidence of ORS and the subsequent development of malignancies have increased. This may be due to the increasing numbers of laparoscopic oophorectomies that are being performed (8,9,11–13).

## 4. Symptoms and clinical features

Abdominal pain due to the compression of adjacent structures by a pelvic mass is the major symptom of ORS. In certain cases, this pelvic mass is only detected incidentally. However, the complaints issued by patients subsequently diagnosed with ORS can vary; pain can present as either cyclic or chronic, and may be described anywhere in the ranges of pressure or a dull ache to severe, sharp or stabbing pains (13,24). Lower back pain, dyspareunia, variable bowel symptoms, pelvic masses or ureteral compression may also be encountered (13,24). In patients with ovarian cancer developing in ovarian remnants, the most common presenting symptom is pain (n=12) (3,6,14–16,25–30). In 3 patients, the presenting sign is a pelvic mass found incidentally during routine check-up (6,15,31).

MR imaging findings and a significantly elevated CA125 level indicate malignant changes, although a normal CA125 level does not preclude the diagnosis. In contrast to the classic presentation of ovarian cancer, ascites does not appear to be an associated feature, as the tumor tends to have a retroperitoneal location (15,16). Blood follicle-stimulating hormone (FSH) levels are useful in confirming the diagnosis of ovarian remnants (13,32), particularly when the levels are in the premenopausal range (<40 mIU/ml) in patients who have undergone BSO. However, as the functioning ovarian tissue that remains is only able to produce estradiol levels that cannot suppress gonadotripin, a FSH level of >40 mIU/ml does not exclude the diagnosis.

## 5. Comments

A predisposing factor for ORS is increased vascularity, which causes hemostasis, endometriosis, pelvic inflammatory disease, pelvic adhesions and altered anatomy, causing difficulties similar to the problems observed with neoplasms and endometriosis (1–9). The most common preexisting conditions for this complication include endometriosis and prior abdominal surgery. Ovarian remnants are commonly encased in adhesions due to the preexisting conditions and prior surgeries. Pain and compression of the adjacent structures are caused by functional changes to the ovarian remnant that result in an increased ovarian volume within a fixed space (33).

Neoplasia may be found within the ovarian remnant tissue. Malignant transformation can also occur within the ovarian remnant tissues. In addition to the case of endometrioid adenocarcinoma described in the original study by Shemwell and Weed (3), other case studies of malignancies discovered in ovarian remnant tissue have been published in the international medical literature (Table I). A case study by Ichigo *et al* (25) described 1 patient from this series, a 48-year-old female who developed a primary ovarian clear cell carcinoma diagnosed through a routine gynecologic examination three years and five months following total abdominal hysterectomy and BSO for endometriosis. This is the first report of a case with a primary ovarian clear cell carcinoma developing in the ovarian remnant. It is important to recognize that unexplored pelvic masses may represent malignant neoplasms, thus forbidding conservative medical management.

Although ORS is a rare condition with an incidence that is difficult to determine, the syndrome can occur following a previous BSO. The majority of cases of patients with ORS are managed by laparotomy, however, the recent literature on predominantly minimally invasive approaches has used the same therapeutic outcome known to produce an excellent outcome. An unexplored pelvic mass following BSO may represent a neoplasia with malignant potential. The risk of the onset of ORS and subsequent malignant involvement may mandate complete surgical resection, which may be the only effective therapy to avoid the recurrence of symptoms. In endometriosis cases, performing a complete excision of the endometriosis and ovarian tissues in the initial surgery can prevent the recurrence of endometriosis, and subsequently the development of ORS and any possible ovarian malignant transformation.

## Figures and Tables

**Table I:** Characteristics of malignant and benign ovarian tumors developing in ovarian remnants.

Case	Age, years	Presenting symptoms	Time to ORS, years	Histological type	Ref.
1	48	Pain	3.5	Clear cell adenocarcinoma	(25)
2	74	Pain	2	Mucinous cystadenocarcinoma	(6)
3	78	Pelvic mass	52	Endometrioid adenocarcinoma	(6)
4	68	Pain	NA	Adenocarcinoma	(26)
5	61	Pain	NA	Adenocarcinoma	(27)
6	69	Pain	NA	Adenocarcinoma	(28)
7	53	Pain	NA	Endometrioid adenocarcinoma	(29)
8	69	Pain	14	Endometrioid adenocarcinoma	(16)
9	67	Pelvic mass	NA	Borderline serous cystadenoma	(31)
10	76	Pain	2	Endometrioid adenocarcinoma	(30)
11	58	Pain	10	Mucinous cystadenocarcinoma	(14)
12	65	Pain	NA	Endometrioid adenocarcinoma	(3)
13–15	51.3 (mean)	Pain (n=2), Pelvic mass (n=1)	NA	Serous cystadenoma (n=3)	(15)
16–22	46.0 (mean)	Pain (n=7)	NA	Simple cyst (n=7)	(34)
23–33	38.6 (mean)	Pain (n=11)	NA	Serous cyst (n=2), simple cyst (n=9)	(10)

ORS, ovarian remnant syndrome; NA, not available.
